# Protocol for the spatiotemporal profiling of RNA within nuclear compartments in human cell lines using SLAM-RT&Tag

**DOI:** 10.1016/j.xpro.2025.104017

**Published:** 2025-08-09

**Authors:** Nadiya Khyzha, Kami Ahmad, Steven Henikoff

**Affiliations:** 1 Basic Sciences Division, Fred Hutchinson Cancer Center, Seattle, WA 98109, USA; 2 Howard Hughes Medical Institute, Chevy Chase, MD 20815, USA

**Keywords:** Bioinformatics, Cell Biology, Gene Expression, Genomics, Molecular Biology, Molecular/Chemical Probes, RNA-seq, Sequence analysis, Sequencing

## Abstract

Measuring RNA residence time within nuclear compartments provides insight into their roles as either storage sites or transient processing hubs. This protocol describes SLAM-RT&Tag, a genomic technique that integrates RNA metabolic labeling with RT&Tag, an RNA proximity labeling method. We detail steps for RNA labeling, library preparation, and computational quantification of T-to-C conversion events to infer RNA dynamics within nuclear compartments in human cell lines.

For complete details on the use and execution of this protocol, please refer to Khyzha et al.^1^

## Highlights

• Steps for quantifying localized RNA dynamics using SLAM-RT&Tag
• Instructions for preparing SLAM-RT&Tag Illumina sequencing libraries
• Procedures for differential kinetic analysis of SLAM-RT&Tag data

## Abstract (editor highlights)

Publisher’s note: Undertaking any experimental protocol requires adherence to local institutional guidelines for laboratory safety and ethics.

## Graphical abstract

**Figure:**
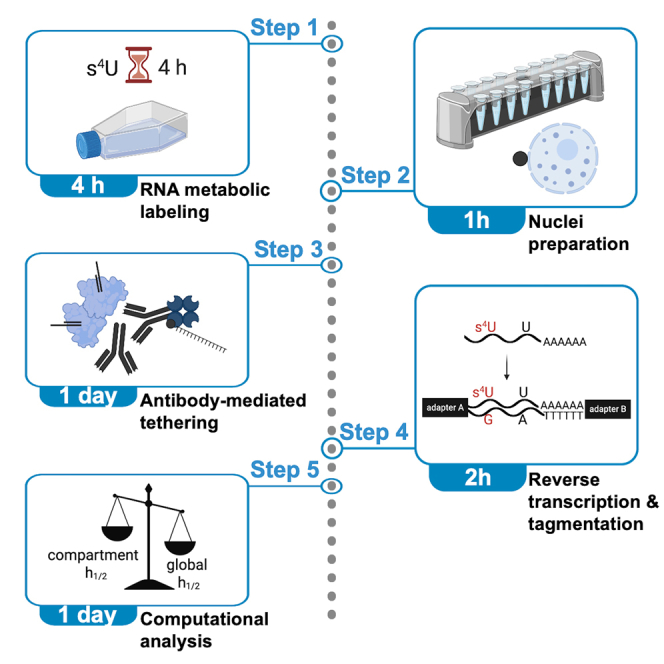

## Before you begin

We previously developed Reverse Transcribe & Tagment (RT&Tag),^2^ a proximity labeling method that leverages antibody-mediated enzyme tethering to capture RNAs in spatial proximity of an epitope. While RT&Tag maps the steady-state RNA populations of nuclear compartments, it does not provide information on RNA turnover. To overcome this limitation, we established SLAM-RT&Tag, which integrates principles of RNA metabolic labeling from SLAM-seq^3^ with RT&Tag to enable spatiotemporal profiling of compartment-associated transcripts.

This protocol describes the application of SLAM-RT&Tag to profile the RNA dynamics within nuclear speckles in human K562 cells. Nuclear speckles are nuclear compartments enriched in polyadenylated RNA and RNA processing factors, which we targeted due to their implication in post-transcriptional splicing.^1^^,^^4^ K562 cells are pulsed with 4-thiouridine (s^4^U) to label nascent RNAs. K562 nuclei are then isolated and bound to paramagnetic beads. The nuclear speckle transcriptome is then captured using an antibody (SC35) targeting the speckle-associated protein, SRRM2.^5^ Biotinylated oligo(dT) and pAG-Tn5 are then tethered, followed by targeted reverse transcription and tagmentation. The s^4^U nucleotides are then misread by the reverse transcriptase, leading to the incorporation of G instead of A in the cDNA strand. This step results in T-to-C mutations when generating Illumina sequencing libraries by PCR amplification. Illumina sequencing libraries are then sequenced and T-to-C conversion events are computationally deduced to quantify half-lives.

While this protocol is demonstrated in K562 cells, it is adaptable to other cell types and nuclear compartments, provided sufficient s^4^U labeling efficiency and nuclei input. The success of SLAM-RT&Tag relies on the use of high-quality, validated antibodies. An IgG antibody should be included as a control to account for global half-lives and serve as a measure of input for the assay.

Incorporation of s^4^U is used to metabolically label newly synthesized RNA. A 4-h s^4^U treatment typically results in 5%–10% labeling efficiency, depending on the nuclear compartment being targeted.^6^ Prolonged s^4^U exposure or pulse-chase experimental designs are not recommended, as s^4^U can impair cell proliferation and induce transcriptome-wide perturbations.^7^ When applying this method to other cell types, the s^4^U labeling conditions will need to be empirically optimized to achieve sufficient incorporation while minimizing effects on gene expression. Lastly, it is essential to include a negative control consisting of unlabeled cells to control for background T-to-C conversion rates.

**CRITICAL:** All steps should be carried out under RNase-free conditions to preserve RNA integrity. Always wear gloves and use RNase-free tubes, tips, and reagents. Thoroughly clean your workbench, pipettes, and equipment with RNAseZap or equivalent RNase decontaminant before beginning the protocol.

### Culture cell lines

**Timing: ∼2–3 days**

1. Seed K562 cells in T-75 flasks containing 15 mL of Iscove’s Modified Dulbecco’s Medium (IMDM) supplemented with 10% fetal bovine serum (FBS). Incubate at 37°C in a 5% CO_2_ humidified incubator.
2. Maintain cells in log-phase growth by passaging every 2–3 days. Keep cultures at a density between 2 x 10^5^ and 1 x 10^6^ cells/mL, ensuring cultures remain sub-confluent.
3. When the cell density reaches approximately 0.7 × 10^6^ cells/mL, proceed with s^4^U labeling.
4. Add s^4^U directly to the culture medium at a final concentration of 100 μM and incubate for 4 h at 37°C in a 5% CO_2_ humidified incubator. Include an unlabeled control in parallel to assess background T-to-C conversion.

**CRITICAL:** Prolonged s^4^U treatments or pulse-chase designs are not recommended, as s^4^U can impair cell proliferation and alter transcriptional profiles.^7^

### Tn5 loading with adapters

**Timing: 2 h**

5. Dilute the mosaic end_Adapter A and mosaic end_reverse oligonucleotides to 200 μM each in Annealing Buffer.
6. In a 1.5 mL RNase-free Eppendorf tube, mix 8 μL of each oligonucleotide.
7. Incubate the oligo mixture at 95°C for 5 min on a heat block to anneal.
8. After incubation, remove the heat block from the heat source and allow the oligo mixture to cool gradually to room temperature (∼45 min, 22°C).
9. Combine the annealed oligonucleotide with 100 μL of 5.5 μM pAG-Tn5 fusion protein.
10. Incubate at room temperature (22°C) for 1 h on a rotating platform to facilitate complex formation.
11. Store the assembled pAG-Tn5–ME-A adapter complex at −20°C until use.

### Antibody conjugation

**Timing: 4 h**

12. Dilute the guinea pig anti-rabbit or rabbit anti-mouse secondary antibody to a final concentration of 1 μg/μL in 90 μL Wash buffer in a 1.5 mL Eppendorf tube.
13. Follow the manufacturer’s instructions. To start, add 10 μL of Modifier Reagent from the Streptavidin Conjugation Kit to the diluted secondary antibody. Mix gently by pipetting.
14. Transfer the antibody–Modifier Reagent mixture to the Streptavidin Mix provided in the kit. Mix thoroughly by pipetting.
15. Incubate at room temperature (22°C) for 3 h in the dark. Wrap the tube in aluminum foil to protect it from light.
16. After incubation, add 10 μL of Quencher Reagent to the conjugation reaction. Mix gently by pipetting.
17. Incubate for an additional 30 min at room temperature (22°C) in the dark.
18. Store the streptavidin-conjugated secondary antibody at 4°C, protected from light. The conjugated antibody is stable for several months under these conditions.

## Key resources table

**Table:** 

REAGENT or RESOURCE	SOURCE	IDENTIFIER
**Antibodies**		

Rabbit anti-IgG (1:100)	Abcam	Cat# ab172730; RRID:AB_2687931
Mouse anti-SC35 (1:100)	Abcam	Cat# ab11826; RRID:AB_298608
Guinea pig anti-rabbit (1:100)	Antibodies Online	Cat# ABIN101961; RRID: AB_10775589
Rabbit anti-mouse (1:100)	Abcam	Cat# ab46540; RRID: AB_2614925

**Chemicals, peptides, and recombinant proteins**		

Hydroxyethyl piperazineethanesulfonic acid (HEPES)	Sigma-Aldrich	Cat# H3375
Potassium chloride (KCl)	Sigma-Aldrich	Cat# P3911
Manganese chloride (MnCl_2_)	Sigma-Aldrich	Cat# M5005
Calcium chloride (CaCl_2_)	Fisher Scientific	Cat# BP510
Triton X-100	Sigma-Aldrich	Cat# X100
Spermidine	Sigma-Aldrich	Cat# S0266
Glycerol	Sigma-Aldrich	Cat# G5516
Sodium chloride (NaCl)	Sigma-Aldrich	Cat# S3014
Ethylenediaminetetraacetic acid (EDTA)	Research Organics	Cat# 3002E
Bovine serum albumin (BSA)	Sigma-Aldrich	Cat# A8577
N-[Tris(hydroxymethyl)methyl]-3-aminopropanesulfonic acid (TAPS)	MilliporeSigma	Cat# T5130
Sodium dodecyl sulfate (SDS)	Sigma-Aldrich	Cat# L4509
Tris(hydroxymethyl)aminomethane	Research Organics Inc.	Cat# 30960T
Ethanol	Fisher Scientific	Cat# 04-355-223
pAG-Tn5	EpiCypher	Cat# 15-1025
4-thiouridine (s^4^U)	Sigma-Aldrich	Cat# T4509
Iodoacetamide (IAA)	Sigma-Aldrich	Cat# I1149
Roche cOmplete mini EDTA free protease inhibitor cocktail	Sigma-Aldrich	Cat# 11836170001
RNasin RNase inhibitor	Promega	Cat# N2515
Concanavalin A (ConA) paramagnetic beads	Bangs Laboratories	Cat# BP531

**Critical commercial assays**		

Iscove’s modified Dulbecco’s medium (IMDM)	ATCC	Cat# 30-2005
Fetal bovine serum (FBS)	Cytiva	Cat# SH30070.03
Streptavidin conjugation kit	Abcam	Cat# ab102921
NEBNext high-fidelity PCR master mix	NEB	Cat# M0541L
Maxima H Minus reverse transcriptase	Thermo Fisher Scientific	Cat# EP0752
High sensitivity D5000 ScreenTape	Agilent Technologies	Cat# 5067-5592
High sensitivity D5000 reagents (sample buffer & ladder)	Agilent Technologies	Cat# 5067-5593
HighPrep PCR cleanup system	MagBio	Cat# AC-60500

**Experimental models: Cell lines**		

Human: K562	ATCC	Cat# CCL-243; RRID: CVCL_0004

**Oligonucleotides**		

Biotinylated-Oligod(T)-MEB: /5Biosg/GTCTCGTGGGCTCGGAGATGTGTA TAAGAGACAGTTTTTTTTTTTTTTTTTT TTTTTTTTTTTTTVN	IDT	NA
Mosaic end_reverse: [PHO]CTGTCTCTTATACACATCT	IDT	NA
Mosaic end_Adapter A: TCGTCGGCAGCGTCAGATGTGTATAAGAGACAG	IDT	NA

**Software and algorithms**		

HISAT2	Kim et al.^8^	http://daehwankimlab.github.io/hisat2/
featureCounts	Liao et al.^9^	https://subread.sourceforge.net/
DESeq2	Love et al.^10^	https://github.com/mikelove/DESeq2
SAMtools	Li et al.^11^	http://www.htslib.org/
SLAM-DUNK	Neumann et al.^6^	https://t-neumann.github.io/slamdunk/
bam2bakR	Schofield et al.^12^	https://github.com/simonlabcode/bam2bakR
bakR	Vock et al.^13^	https://github.com/simonlabcode/bakR
RStudio v.4.1.1	R Project	https://www.r-project.org/

**Other**		

0.2 ml PCR 8 strip magnetic separator	Permagen Labware	Cat# MSRLV08
MACSiMAG separator	Miltenyi Biotec	Cat# 130-092-168
PCR Rigid 8-tube strips with individually attached flat caps, clear, 0.2 mL	BrandTech	Cat# P1200
250 μL BioClean Ultra wide-orifice, low retention	RAININ	Cat# 30389250
1,000 μL BioClean Ultra wide-orifice, low retention	RAININ	Cat# 30389221
TapeStation 4200 electrophoresis system	Agilent Technologies	Cat# G2991A

## Materials and equipment

Recipes for the solutions and buffers used in this protocol are described and listed below. Ensure that all the buffers, reagents, and equipment are made with RNase-free water.

**Table:** Annealing buffer

Reagent	Final concentration	Amount
1 M Tris pH 8.0	10 mM	100 μL
5 M NaCl	50 mM	100 μL
0.5 M EDTA	1 mM	20 μL
RNase-free water	N/A	9780 μL
**Total**	**N/A**	**10 mL**

Store at room temperature (22°C) for up to 6 months.

**Table:** Binding buffer

Reagent	Final concentration	Amount
1 M HEPES-KOH pH 7.9	20 mM	200 μL
1 M KCl	10 mM	100 μL
1 M CaCl_2_	1 mM	10 μL
1 M MnCl_2_	1 mM	10 μL
RNase-free water	N/A	9680 μL
**Total**	**N/A**	**10 mL**

Store at room temperature (22°C) for up to 6 months.

**Table:** NE1 buffer

Reagent	Final concentration	Amount
1 M HEPES-KOH pH 7.9	20 mM	1 mL
1 M KCl	10 mM	0.5 mL
10% Triton X-100	0.10%	0.5 mL
100% Glycerol	20%	10 mL
2 M spermidine	0.5 mM	12.5 μl
Roche Complete Protease Inhibitor EDTA-Free Tablet	N/A	1 tablet
RNase-free water	N/A	38 mL
**Total**	**N/A**	**50 mL**

Store at room temperature (22°C) for up to 6 months. Add the spermidine and protease inhibitor cocktail to the buffer before use, after which store at 4°C for up to 1 week.

**Table:** Wash buffer

Reagent	Final concentration	Amount
1 M HEPES-KOH pH 7.5	20 mM	1 mL
5 M NaCl	150 mM	1.5 mL
2 M spermidine	0.5 mM	12.5 μL
Roche Complete Protease Inhibitor EDTA-Free Tablet	N/A	1 tablet
RNase-free water	N/A	47.5 mL
**Total**	**N/A**	**50 mL**

Store at room temperature (22°C) for up to 6 months. Add the spermidine and protease inhibitor cocktail to the buffer before use, after which store at 4°C for up to 1 week.

**Table:** Antibody buffer

Reagent	Final concentration	Amount
0.5 M EDTA	2 mM	8 μL
30% BSA	0.10%	6.7 μL
Wash Buffer	N/A	1985.3 μL
**Total**	**N/A**	**2 mL**

Store at 4°C for up to 1 week.

**Table:** 300Wash buffer

Reagent	Final concentration	Amount
1 M HEPES-KOH pH 7.5	20 mM	1 mL
5 M NaCl	300 mM	3 mL
2 M spermidine	0.5 mM	12.5 ul
Roche Complete Protease Inhibitor EDTA-Free Tablet	N/A	1 tablet
RNase-free water	N/A	46 mL
**Total**	**N/A**	**50 mL**

Store at room temperature (22°C) for up to 6 months. Add the spermidine and protease inhibitor cocktail to the buffer before use, after which store at 4°C for up to 1 week.

**Table:** Post-tagmentation buffer

Reagent	Final concentration	Amount
1 M TAPS pH 8.5	10 mM	10 μL
RNase-free water	N/A	990 μL
**Total**	**N/A**	**1 mL**

Make fresh directly before use.

**Table:** SDS release buffer

Reagent	Final concentration	Amount
1 M TAPS pH 8.5	10 mM	10 μL
10% SDS	0.10%	10 μL
RNase-free water	N/A	980 μL
**Total**	**N/A**	**1 mL**

Make fresh directly before use.

**Table:** Neutralization buffer

Reagent	Final concentration	Amount
10% Triton X-100	0.67%	67 μL
RNase-free water	—	933 μL
**Total**	**N/A**	**1 mL**

Make fresh directly before use.

## Step-by-step method details

### Activation of Concanavalin A-coated magnetic beads

**Timing: 10 min**

Concanavalin A (ConA)-coated magnetic beads are used to immobilize nuclei. This step activates the ConA beads using divalent cations (Ca^2+^ and Mn^2+^) and prepares them for nuclei binding.

1. Thoroughly mix the ConA bead slurry by inverting the tube several times to ensure a uniform suspension.
2. Aliquot 5 μL of beads per reaction into a 1.5 mL Eppendorf tube using wide-bore 200 μl tips (e.g., 40 μL total for 8 samples).
3. Add 1 mL of Binding Buffer to the bead suspension. The beads will disperse upon the addition of Binding Buffer.
4. Place the tube on a magnetic stand for ∼1 min until the solution clears. Carefully remove and discard the supernatant.
5. Repeat the wash with 1 mL fresh Binding Buffer.
6. Resuspend the beads in 5 μL of Binding Buffer per reaction (e.g., 40 μL total).
7. Aliquot 5 μL of activated ConA beads into each well of an 8-tube PCR strip.

**CRITICAL:** ConA beads settle rapidly after mixing. Work quickly to ensure even distribution across samples. Use of wide-bore pipette tips is necessary to enable easier and more consistent pipetting of viscous bead suspensions and prevent tip clogging.

### Isolation of nuclei from human K562 cells

**Timing: 1 h**

K562 nuclei are isolated using a gentle lysis buffer to preserve nuclear integrity for downstream tethering steps.

8. Collect 4 × 10^6^ K562 cells per reaction in a 15 mL Falcon tube.
9. Centrifuge at 600 × *g* for 5 min at room temperature (22°C).
10. Aspirate and discard the media.
11. Resuspend the cell pellet in 1 mL of ice-cold Wash Buffer supplemented with a 1:40 dilution of RNase inhibitor using a 1 mL wide-bore pipette tip.
12. Centrifuge at 600 × *g* for 5 min at room temperature (22°C).
13. Carefully remove the supernatant.
14. Resuspend the cell pellet in 500 μL of NE1 buffer supplemented with 1:40 RNase inhibitor using a 1 mL wide-bore pipette tip.
15. Incubate on ice for 10 min to lyse cells and release nuclei.
16. Centrifuge at 600 × *g* for 5 min at room temperature (22°C).
17. Carefully remove the supernatant. The nuclei pellet will be transparent and difficult to see.
18. Resuspend the nuclei pellet in 1 mL of Wash Buffer supplemented with 1:40 RNase inhibitor using a 1 mL wide-bore pipette tip.
19. Distribute 50 μL of resuspended nuclei (approx. 200,000 nuclei) into each PCR tube containing 5 μL of pre-activated ConA beads using a 200 μL wide-bore pipette tip. Mix gently by pipetting up and down.
20. Incubate at room temperature (22°C) for 10 min to allow the nuclei to bind to the conA beads.

**CRITICAL:** It is advised to use wide-bore pipette tips when handling nuclei suspensions to minimize shear stress and prevent damage to nuclei during pipetting.

**CRITICAL:** Using the appropriate number of nuclei per reaction is essential. Excess nuclei can cause bead stickiness and sample loss. Meanwhile, too few nuclei can result in excessive primer artifact in the final sequencing library. We strongly recommend performing a nuclei titration to determine the optimal input for your specific cell type.

**CRITICAL:** It is imperative to supplement buffers with spermidine, which provides the positive charge necessary to maintain the integrity of nuclear speckles.

### Iodoacetamide treatment for s^4^U carboxyamidomethylation

**Timing: 1 h**

Iodoacetamide is used to alkylate the thiol groups on s^4^U, resulting in carboxyamidomethylation. The treatment is performed early in the protocol to avoid potential denaturation of antibodies or the pAG-Tn5 fusion protein.

21. Prepare the Iodoacetamide Reaction Master Mix by combining the following per reaction in a 1.5 mL Eppendorf tube:

**Table:** 

Reagent	Amount
Wash Buffer	38.75 μL
DMSO	5 μL
100 mM Iodoacetamide	5 μL
RNase Inhibitor	1.25 μL

22. Vortex the master mix for a few seconds and spin down to collect contents.
23. Place the PCR strip tubes on a magnetic stand. Allow ∼1 min for ConA beads to clear from the solution. Carefully remove and discard the supernatant from the nuclei-binding step.
24. Add 50 μL of Iodoacetamide Reaction Master Mix to each PCR tube. Mix by gently flicking each tube.
25. Incubate at 37°C for 1 h in a thermocycler.

### Primary antibody binding

**Timing: 2 h 15 min**

Primary antibodies targeting an epitope enriched within a nuclear compartment are incubated with ConA-bound nuclei.

26. Prepare the Primary Antibody Master Mix by combining 50 μL of Antibody Buffer supplemented with a 1:40 dilution of RNase inhibitor per reaction and a 1:100 dilution of the primary antibody for each primary antibody used in separate 1.5 mL Eppendorf tubes.
27. Vortex the master mixes for a few seconds and spin down to collect contents.
28. Place the PCR strip tubes on a magnetic stand. Allow ∼1 min for ConA beads to clear from solution. Carefully remove and discard the supernatant from the iodoacetamide treatment step.
29. Add 200 μL of Wash Buffer to each PCR tube. Place the tubes back on the magnetic stand and allow ∼1 min for beads to separate. Discard supernatant.
30. Remove the PCR strip from the magnetic stand and repeat the wash with 200 μL of fresh Wash Buffer.
31. Add 50 μL of the Primary Antibody Master Mix to each PCR tube. Mix by gently flicking each tube.
32. Incubate on a nutator at room temperature (22°C) for 2 h or in the fridge (4°C) overnight (16 h).

***Note:*** Antibody concentrations may require optimization depending on target abundance. A 1:100 dilution is recommended as a starting point.

**Pause point:** Tubes can be stored in Antibody Master Mix overnight (16 h) at 4°C following primary antibody incubation.

### Streptavidin-conjugated secondary antibody binding

**Timing: 1 h**

A streptavidin-conjugated secondary antibody is used to amplify the signal and introduce streptavidin binding sites for the subsequent tethering of biotinylated oligos. This enhances capture efficiency and facilitates robust recruitment of the pAG-Tn5 fusion protein.

33. Prepare the Secondary Antibody Master Mix by combining 50 μL Wash Buffer supplemented with 1:40 RNase inhibitor per reaction and 1:100 dilution of streptavidin-conjugated secondary antibody for each secondary antibody used in separate 1.5 mL Eppendorf tubes.
34. Vortex the master mixes for a few seconds and spin down to collect contents.
35. Place the PCR strip tubes on a magnetic stand. Allow ∼1 min for ConA beads to clear from solution. Carefully remove and discard the supernatant from the primary antibody binding step.
36. Add 50 μL of the Secondary Antibody Master Mix to each PCR tube. Mix by gently flicking the tubes.
37. Incubate on a nutator at room temperature (22°C) for 1 h.

**Pause point:** Tubes can be stored in Secondary Antibody Master Mix overnight (16 h) at 4°C following secondary antibody incubation.

### Biotinylated Oligo(dT)-ME-B binding

**Timing: 30 min**

Biotinylated Oligo(dT)-ME-B is bound to the streptavidin-conjugated secondary antibody to target polyadenylated RNAs for reverse transcription later.

38. Prepare the Oligo(dT) Master Mix by combining 50 μL Wash Buffer supplemented with 1:40 RNase inhibitor per reaction and a 1:50 dilution of Biotinylated Oligo(dT)-ME-B (final concentration 0.25 μM) in a 1.5 mL Eppendorf tubes.
39. Vortex the master mixes for a few seconds and spin down to collect contents.
40. Place the PCR strip tubes on a magnetic stand. Allow ∼1 min for ConA beads to clear from solution. Carefully remove and discard the supernatant from the streptavidin-conjugated secondary antibody binding step.
41. Add 200 μL Wash Buffer to each PCR tube. Place the tubes back on the magnetic stand, wait ∼1 min, and discard the supernatant.
42. Remove from magnetic stand. Repeat the wash with an additional 200 μL Wash Buffer as above.
43. Add 50 μL of the Oligo(dT) Master Mix to each PCR tube. Mix by gently flicking the tubes.
44. Incubate on a nutator at room temperature (22°C) for 20 min.

#### Protein AG-Tn5 binding

**Timing: 1 h 15 min**

Protein AG-Tn5 (pAG-Tn5) is targeted to the antibody-tethered sites via the primary and streptavidin-conjugated secondary antibodies. This step enables spatially targeted tagmentation of RNA–cDNA hybrids in later steps. A high-salt 300Wash Buffer is used to minimize off-target binding of pAG-Tn5 to accessible chromatin.

45. Prepare the pAG-Tn5 Master Mix by combining 50 μL 300Wash Buffer supplemented with 1:40 RNase inhibitor per reaction and 1:200 dilution of pAG-Tn5 loaded with ME-A in a 1.5 mL Eppendorf tube.
46. Vortex the master mixes for a few seconds and spin down to collect contents.
47. Place the PCR strip tubes on a magnetic stand. Allow ∼1 min for ConA beads to clear from solution. Carefully remove and discard the supernatant from the Oligo(dT) binding step.
48. Add 200 μL Wash Buffer to each PCR tube. Place the tubes back on the magnetic stand, wait ∼1 min, and discard the supernatant.
49. Remove from magnetic stand. Repeat the wash with an additional 200 μL Wash Buffer as above.
50. Add 50 μL of the pAG-Tn5 Master Mix to each PCR tube. Mix by gently flicking the tubes.
51. Incubate on a nutator at room temperature (22°C) for 1 h.

***Note:*** The optimal dilution of pAG-Tn5 may vary based on the activity of the specific pAG-Tn5 preparation. We recommend titrating empirically for best results.

***Note:*** If using pAG-Tn5 purified in-house, test for RNase contamination as *E. coli*-derived RNases may co-purify. Conventional RNase inhibitors typically do not inhibit bacterial RNases. We recommend supplementing with SUPERase·In (Fisher Scientific AM2694) for broader protection.

#### Reverse transcription and tagmentation

**Timing: 2 h 15 min**

Perform reverse transcription and tagmentation simultaneously. Both the reverse transcriptase and the pAG-Tn5 are Mg^2+^ dependent and have compatible buffers.

52. Prepare the Reverse Transcription Reaction Master Mix by combining the following per reaction in a 1.5 mL Eppendorf tube:

**Table:** 

Reagent	Amount
5x Reverse Transcription Buffer	4 μL
dNTP Mix (10 mM)	1 μL
Maxima Reverse Transcriptase	1 μL
RNase Inhibitor	0.5 μL
ddH_2_O	13.5 μL

53. Vortex the master mixes for a few seconds and spin down to collect contents.
54. Place the PCR strip tubes on a magnetic stand. Allow ∼1 min for ConA beads to clear from solution. Carefully remove and discard the supernatant from the pAG-Tn5 binding step.
55. Add 200 μL 300Wash Buffer to each PCR tube. Place the tubes back on the magnetic stand, wait ∼1 min, and discard the supernatant.
56. Remove from the magnetic stand. Repeat the wash with an additional 200 μL 300Wash Buffer as above.
57. Add 20 μL of the Reverse Transcription Master Mix to each PCR tube. Mix by gently flicking the tubes.
58. Incubate at 37°C for 2 h in a thermocycler.

#### pAG-Tn5 release from RNA-cDNA hybrids

**Timing: 1 h 15 min**

The pAG-Tn5 is released from RNA-cDNA hybrids using SDS and heat. This step allows for efficient library amplification in the downstream PCR reaction.

59. Place the PCR strip tubes on a magnetic stand. Allow ∼1 min for ConA beads to clear from solution. Carefully remove and discard the supernatant from the reverse transcription and tagmentation step.
60. Add 50 μL Post-tagmentation Wash Buffer to each PCR tube. Place the tubes back on the magnetic stand, wait ∼1 min, and discard the supernatant.
61. Add 5 μL SDS Release Buffer to each PCR tube. Mix by gently flicking the tubes.
62. Incubate at 58°C for 1 h in a thermocycler.

***Note:*** Do not pipette the samples after adding SDS Release Buffer. The bead solution becomes sticky and can adhere to pipette tips, potentially leading to sample loss.

#### Generation of Illumina sequencing libraries by PCR

**Timing: 45 min**

Illumina sequencing libraries are generated by PCR amplification using primers that anneal to the ME sequences inserted by pAG-Tn5 and the biotinylated oligo(dT).

63. Add 15 μL of Neutralization Buffer to each PCR tube. Mix by gently flicking the tubes.
64. Prepare the PCR Reaction by combining the following per reaction in a 1.5 mL Eppendorf tube:

**Table:** 

Reagent	Amount
I7 primer	2 μL
I5 primer	2 μL
NEBNext High-Fidelity PCR Master Mix	25 μL

65. Add 29 μL of the PCR Reaction to each PCR tube. Mix by vortexing for a few seconds and spinning down to collect contents.
66. Perform PCR using the following cycle conditions:

**Table:** 

Steps	Temperature	Time	Cycles
Gap Repair	58°C	5 min	1
	72°C	5 min	1
Initial Denaturation	98°C	30 sec	1
Denaturation	98°C	10 sec	14 cycles
Annealing/ Extension	60°C	10 min	
Final extension	72°C	1 min	1
Hold	4°C	forever	

#### Library bead cleanup

**Timing: 30 min**

Purify PCR-amplified libraries using AMPure XP beads to remove primer dimers, adapter artifacts, and other small contaminants.

67. Mix 50 μL of the PCR reaction with 40 μL of AMPure XP beads in each PCR tube. Vortex for a few seconds and spin down to collect.
68. Incubate the bead mixture at room temperature (22°C) for 5–10 min.
69. Place the PCR tubes on a magnetic rack. Allow ∼2 min for beads to gather. Carefully remove and discard the supernatant.
70. Wash the beads twice by adding 200 μL of freshly prepared 80% ethanol while the tubes remain on the magnetic rack. After each wash, remove and discard the ethanol carefully.
71. Centrifuge the tubes for a few seconds to collect any residual ethanol. Place back on the magnetic rack and remove any remaining ethanol using a 20 μL pipette tip.
72. Allow beads to dry for ∼1 min to remove any residual ethanol.
73. Remove tubes from the magnetic rack. Add 22 μL of 10 mM Tris-HCl to each tube to resuspend the beads. Vortex for a few seconds, spin down, and incubate at room temperature (22°C) for 5–10 min.
74. Place the tubes back on the magnetic rack. Once the solution clears, transfer the supernatant containing the purified library to a new clean tube.

***Note:*** Do not allow the beads to over dry after ethanol removal, as this can reduce DNA recovery.

#### Tapestation analysis

**Timing: 20 min**

Assess the size distribution and quantity of the amplified libraries using a High Sensitivity D5000 Tapestation assay.

75. Prepare samples for the Tapestation by combining 2 μL of library sample with 2 μL of sample dye in PCR strip tubes. Include one tube containing 2 μL of ladder and 2 μL of sample dye.
76. Thoroughly vortex the Tapestation samples and spin down to collect.
77. Run the Tapestation with a High Sensitivity D5000 Tapestation chip according to the manufacturer’s instructions.
78. Check the library size distribution, which should range between 200–700 bp.

***Note:*** If a large peak around 150 bp is observed, it likely represents adapter artifacts. Perform an additional round of AMPure XP bead clean-up to remove these artifacts.

#### Deep sequencing and data processing

**Timing: ∼1 week**

SLAM-RT&Tag libraries require approximately 20–30 million single-end raw reads per sample for accurate data interpretation.^6^ This read depth ensures sufficient coverage for identifying differentially enriched transcripts within a compartment and quantifying RNA half-lives.

79. Download an hg19 genome assembly file (FASTA) from UCSC (https://hgdownload.soe.ucsc.edu/goldenPath/hg19/bigZips/hg19.fa.gz) and GENCODE GRCh37.p13 v.19 gene transfer format (GTF) file (https://ftp.ebi.ac.uk/pub/databases/gencode/Gencode_human/release_19/gencode.v19.annotation.gtf.gz).
80. Use single-end raw reads (FASTQ files) from unlabeled samples to define transcripts enriched in compartment of interest, nuclear speckles in this example.
  a. Build an hg19 genome assembly index using HISAT2^8^ by running the following command:
    > hisat2-build PATH/hg19.fa hg19
  b. Align raw reads to the hg19 genome assembly using HISAT2^8^ and generating a BAM (binary alignment map) using SAMtools.^11^ Accomplish this by running the following command:
    > hisat2 --max-intronlen 30000 --rna-strandness F --summary-file input_name_HISAT2_summary -x PATH/hg19 -U input_unlabeled.fastq.gz | samtools view -S -b > output.bam
  c. Count the number of aligned reads in BAM files that overlap with the reference transcriptome GTF file using the featureCounts^9^ tool from Subread by running the following command:
    > featureCounts -s 1 -t exon -g gene_id -a hg19.gtf -o sample_name.exon.tabular input.bam
  d. Perform differential enrichment analysis using DESeq2^10^ in R studio. Include two conditions (IgG and SC35) with 3 biological replicates for each condition. Use the following commands:
    # Load the necessary packages
    library(rtracklayer)
    library(DESeq2)
    # Load gtf file
    gtf<- import("PATH/hg19.gtf")
    gtf_df<-as.data.frame(gtf)
    gtf_df<-(gtf_df[gtf_df$type=="gene",])[,c(1:6,10,12,14)]
    # Load featureCounts count tables
    featureCounts_IgG_n1<-read.delim("PATH/featureCounts/IgG_n1.exon.tabular", comment.char="#")[,c(1,7)]
    featureCounts_SC35_n1<- read.delim("PATH/featureCounts/SC35_n1.exon.tabular", comment.char="#")[,c(1,7)]
    featureCounts_IgG_n2<- read.delim("PATH/featureCounts/IgG_n2.exon.tabular", comment.char="#")[,c(1,7)]
    featureCounts_SC35_n2<- read.delim("PATH/featureCounts/SC35_n2.exon.tabular", comment.char="#")[,c(1,7)]
    featureCounts_IgG_n3<- read.delim("PATH/featureCounts/IgG_n3.exon.tabular", comment.char="#")[,c(1,7)]
    featureCounts_SC35_n3<- read.delim("PATH/featureCounts/SC35_n3.exon.tabular", comment.char="#")[,c(1,7)]
    # Assemble featureCount count tables into a matrix
    Counts<- cbind(featureCounts_IgG_n1[,2], featureCounts_SC35_n1[,2],
      featureCounts_IgG_n2[,2], featureCounts_SC35_n2[,2],
      featureCounts_IgG_n3[,2], featureCounts_SC35_n3[,2])
    colnames(Counts)<-c("IgG n1", "SC35 n1", "IgG n2", "SC35 n2", "IgG n3", "SC35 n3")
    rownames(Counts)<-featureCounts_IgG_n1[,1]
    # Assemble metadata for the featureCount count table matrix
    Protein<- c("IgG", "SC35", "IgG", "SC35", "IgG", "SC35")
    Condition<- c("n1","n1", "n2","n2", "n3","n3")
    Metadata<- cbind(Protein, Condition)
    rownames(Metadata)<- c("IgG n1", "SC35 n1","IgG n2", "SC35 n2", "IgG n3", "SC35 n3")
    # Perform DESeq normalization
    Count_Table <- DESeqDataSetFromMatrix(countData = Counts, colData = Metadata,
        design = ∼ Protein + Condition)
    Count_Table_DESeq<- DESeq(Count_Table)
    Counts_normalized<- cbind(gtf_df,counts(Count_Table_DESeq, normalized=TRUE))
    # Function used to retrieve differentially enriched transcripts
    Retrieve <- function(n1, n2, n3, n4) {
      Results<-results(n4, contrast= c("Protein", n1, n2), name = n3)
      Results_log2<-Results$log2FoldChange
      Results_pvalue<-Results$padj
      diffexpressed <- "NO"
      Results_combined<- cbind(gtf_df, Results_log2, Results_pvalue, diffexpressed)
      Results_combined<- Results_combined[!is.na(Results_log2),]
      Results_combined<-as.data.frame(Results_combined)
      Results_combined$diffexpressed[Results_combined$Results_log2 >1 &
        Results_combined$Results_pvalue <0.05] <- "UP"
      Results_combined$diffexpressed[Results_combined$Results_log2 < -1 &
        Results_combined$Results_pvalue <0.05] <- "DOWN"
      Results_combined<-Results_combined[Results_combined$diffexpressed != "NO",]
      return(Results_combined)
    }
    # Use the function above to call differentially enriched transcripts and subdivide based on enriched or depleted
    Differentially_enriched_UPDOWN <- Retrieve("SC35", "IgG", "Protein_SC35_vs_IgG", Count_Table_DESeq)
    Differentially_enriched_UP <- Differentially_enriched_UPDOWN[(Differentially_enriched_UPDOWN$diffexpressed=="UP"),]
    Differentially_enriched_DOWN <- Differentially_enriched_UPDOWN[(Differentially_enriched_UPDOWN$diffexpressed=="DOWN"),]
81. Process the single-end raw reads from unlabeled and s^4^U labeled samples using the SlamDunk^6^ pipeline (https://t-neumann.github.io/slamdunk/) to perform T-to-C mutation-aware alignment and estimate the percentage of T-to-C mutations.
  a. Run the following command to run the SlamDunk pipeline:

> slamdunk all -r PATH/hg19.fa -b PATH/hg19_gtf.bed -o aligned -t 12 PATH/fastq_file.gz

***Note:*** Review the SLAMDunk summary output files to assess the T-to-C conversion efficiency. The expected conversion rate should be approximately 5%–10%. A low conversion rate may indicate insufficient s^4^U labeling or inefficient iodoacetamide treatment.

82. Sort the filtered BAM (binary alignment map) files produced by the SlamDunk pipeline using SAMtools^11^ sort command:

> samtools sort -n -o output_BAM_File.bam input_BAM_File.bam

83. Run the bam2bakR^12^ Snakemake pipeline (https://github.com/simonlabcode/bam2bakR) to generate a .cB file necessary for downstream analysis with BakR.
  a. Prepare the following input files: sorted BAM files from SlamDunk, a genome assembly file (FASTA), a gene transfer format (GTF) file, and a .yaml configuration file.
  b. Follow installation instructions (https://github.com/simonlabcode/bam2bakR) to set-up the bam2bakR conda environment. Establish the environment using the following commands:
    > conda activate deploy_snakemake
    > snakedeploy deploy-workflow https://github.com/isaacvock/fastq2EZbakR.git --branch main
  c. Set the .yaml configuration file as shown below, modifying the sample information to match the fastq files used in the SlamDunk step:
    ######## Parameters you MUST set #########
    # location of ALL (including -s4U control) bam files samples:
      WT_ctl_1: PATH/WT_ctl_1.bam
      KO_ctl_1: PATH/KO_ctl_1.bam
      WT_4SU_1: PATH/WT_4SU_1.bam
      KO_4SU_1: PATH/KO_4SU_1.bam
      WT_ctl_2: PATH/WT_ctl_2.bam
      KO_ctl_2: PATH/KO_ctl_2.bam
      WT_4SU_2: PATH/WT_4SU_2.bam
      KO_4SU_2: PATH/KO_4SU_2.bam
    # sample IDs of -s4U control bam files (or leave blank if you have none)
    # Needs to be same sample ID(s) that show up under samples entry
    control_samples: ['WT_ctl_1', 'KO_ctl_1', 'WT_ctl_2', 'KO_ctl_2']
    # location of annotation gtf file
    annotation: PATH/hg19.gtf
    # location of genome fasta file
    genome_fasta: PATH/hg19.fa
    ######## Parameters you should probably double check ########
    # Number of cpus to be used by pipeline
    cpus: 4
    # Format of reads
    FORMAT: "SE" # (PE, SE, NU)
      # [SE - single end reads]
      # [NU - including non-unique] (not tested)
    # Are you using the Windows subsystem for linux? 0 = Yes, 1 = No
    WSL: 1
    # Number of reads per fragment when splitting up bam file
    fragment_size: 3500000
    # Type of mutations of interest
    mut_tracks: "TC" # ("TC", "GA", "TC,GA")
    # whether to make .tdf files or not
    make_tracks: True
    # Minimum base quality to call mutation
    minqual: 40
    # Which columns to keep in final cB.csv.gz file
    keepcols: "sample,sj,io,ei,ai,GF,XF,rname"
    # String common to spike-in gene_ids in annotation gtf
     # If you have no spike-ins, then this should be "∖"∖"", i.e., a string containing ""
    spikename: "∖"∖""
    # If True, tracks will be normalized
    normalize: True
    ######## Parameters you should NEVER alter ########
    awkscript: workflow/scripts/fragment_sam.awk
    mutcall: workflow/scripts/mut_call.py
    mutcnt: workflow/scripts/count_triple.py
    count2tracks: workflow/scripts/count_to_tracks.py
  d. Run the bam2bakR Snakemake workflow with the following commands:
    > conda activate complete_pipeline
    > snakemake --cores 8
  e. Load the .tdf output files into IGV (Integrative Genomics Viewer) to visualize the SLAM-RT&Tag signal. Tracks are separated based on reads containing between 0 and 5 numbers of T-to-C mutations per read.
84. Perform differential kinetic analysis using the BakR^13^ Bioconductor package (https://github.com/simonlabcode/bakR). BakR performs several critical analyses, including estimating RNA degradation rate constants and identifying changes in RNA stability across different experimental conditions.

# Load dependencies

library(dplyr)

library(bakR)

library(purrr)

# Loading the cB data

cB_data <- read.csv("/PATH/cB.csv")

cB_sub <- cB_data[,c(5, 1, 10, 2,11)]

# Setting up the bakRData objects for pulse conditions

cB_pulse<- cB_sub[(cB_sub$sample=="KO_4SU_1")|(cB_sub$sample=="KO_4SU_2")| (cB_sub$sample=="WT_4SU_1")|(cB_sub$sample=="WT_4SU_2")| (cB_sub$sample=="KO_ctl_1")|(cB_sub$sample=="KO_ctl_2")|

(cB_sub$sample=="WT_ctl_1")|(cB_sub$sample=="WT_ctl_2"),]

tl<- vector(length=8)

tl<- c(4,4,0,0,4,4,0,0)

Exp_ID<- vector(length=8)

Exp_ID<- c(2,2,2,2,1,1,1,1)

metadf<- cbind(tl, Exp_ID)

rownames(metadf) <- c("KO_4SU_1", "KO_4SU_2", "KO_ctl_1", "KO_ctl_2",

  "WT_4SU_1", "WT_4SU_2", "WT_ctl_1", "WT_ctl_2")

data_pulse<- bakRData(cB_pulse, metadf)

# Perform bakRFit using the Fast_Fit parameter first

fit_pulse <- bakRFit(data_pulse, NSS= FALSE, totcut=50)

# Visualize differential kinetic analysis of transcripts using a volcano plot

plotVolcano(fit_pulse$Fast_Fit)

# Follow up with bakRFit using the more stringent Hybrid_Fit parameter

fit_pulse <- bakRFit(fit_pulse, HybridFit = TRUE, NSS = FALSE)

# visualize differential kinetic analysis of transcripts using a volcano plot

plotVolcano(fit_pulse$Hybrid_Fit)

# Retrieve differential kinetic analysis

 dka_info <- fit_pulse[["Hybrid_Fit"]][["Effects_df"]]

85. Retrieve degradation rate constants (k_deg_) and half-lives for individual transcripts using the following script:

# Retrieve global and localized degradation rate constants

 kdeg_info <- fit_pulse[["Hybrid_Fit"]][["Kdeg_df"]]

# Divide into global (IgG) and localized (SC35) groups

 kdeg_info_global <- kdeg_info[kdeg_info$Exp_ID==1,]

 kdeg_info_localized <- kdeg_info[kdeg_info$Exp_ID==2,]

# Convert degradation rate constants into half-lives using the following formula

 kdeg_info_global$half_lives <- 0.69314718056/kdeg_info_global$kdeg

 kdeg_info_localized$half_lives <- 0.69314718056/kdeg_info_localized$kdeg

## Expected outcomes

### Tapestation trace of SLAM-RT&Tag libraries

**Figure 1:**
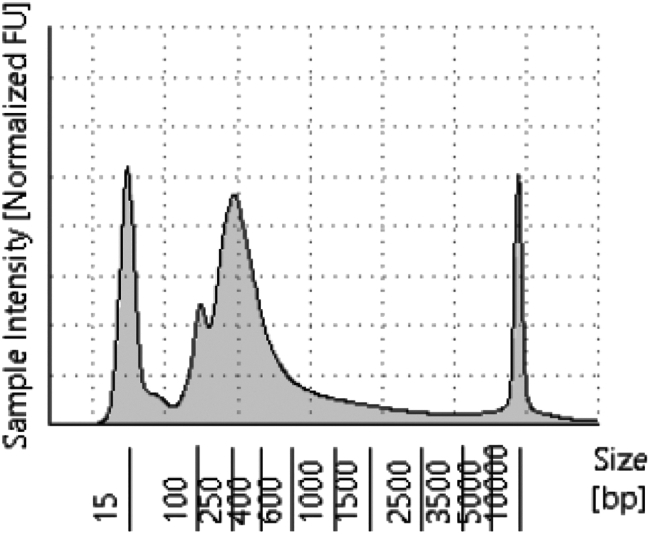
Representative Tapestation trace of a successful SLAM-RT&Tag library

Before sequencing, SLAM-RT&Tag libraries should be analyzed using a Tapestation assay to assess their size distribution and yield. A successful Tapestation trace (Figure 1) will show a dominant peak between 200–700 bp, representing the amplified library. A smaller peak near 150 bp, typically due to primer artifact, will be present but should be lower in abundance than the main library peak. Failure to detect a clear library peak or the presence of excessive primer artifact suggests experimental failure.

### Alignment statistics of SLAM-RT&Tag libraries

**Figure 2:**
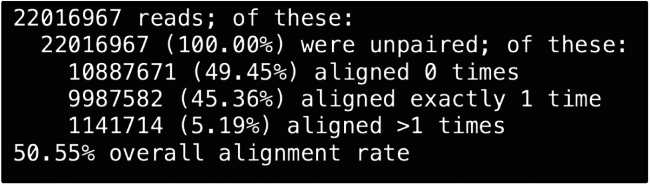
Representative HISAT2 alignment summary statistics

Reads from unlabeled SLAM-RT&Tag libraries are aligned to the genome using HISAT2. Typically, 40%–50% of reads are uniquely mapped, with a minor percentage of multimapping reads (Figure 2). A low mapping rate may indicate poor library quality, often due to the sequencing of adapter artifacts.

We do not recommend using HISAT2 to align SLAM-RT&Tag libraries from s^4^U-labeled cells, as it is not T-to-C mutation-aware. Instead, use the SlamDunk pipeline, which is optimized for aligning reads containing T-to-C conversions. Similar to HISAT2, an overall alignment rate of ∼40%–50% is expected. A substantially lower mapping rate may indicate excessive sequencing of adapter artifacts.

### Expected T-to-C mutation rate

**Figure 3:**
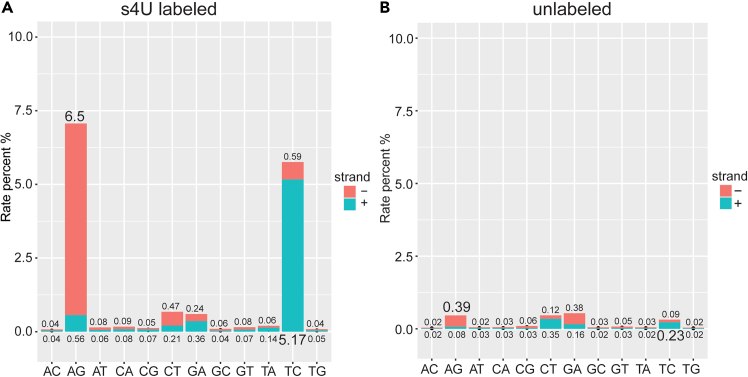
Representative SLAM-DUNK summary outputs Stacked bargraphs show the percentage of mutation types on each strand in (A) s^4^U-labeled cells and (B) unlabeled cells.

Robust T-to-C conversion is critical for accurate differential kinetic analysis. In labeled samples, the SlamDunk pipeline typically detects T-to-C mutation rates of ∼5%–10% (Figure 3A), whereas background rates in unlabeled controls remain below 0.5% (Figure 3B). If conversion rates fall below 5%, consider optimizing the s^4^U labeling duration or concentration.

### Expected SLAM-RT&Tag signal over the gene bodies of *MALAT1* and *MAN2C1*

**Figure 4:**
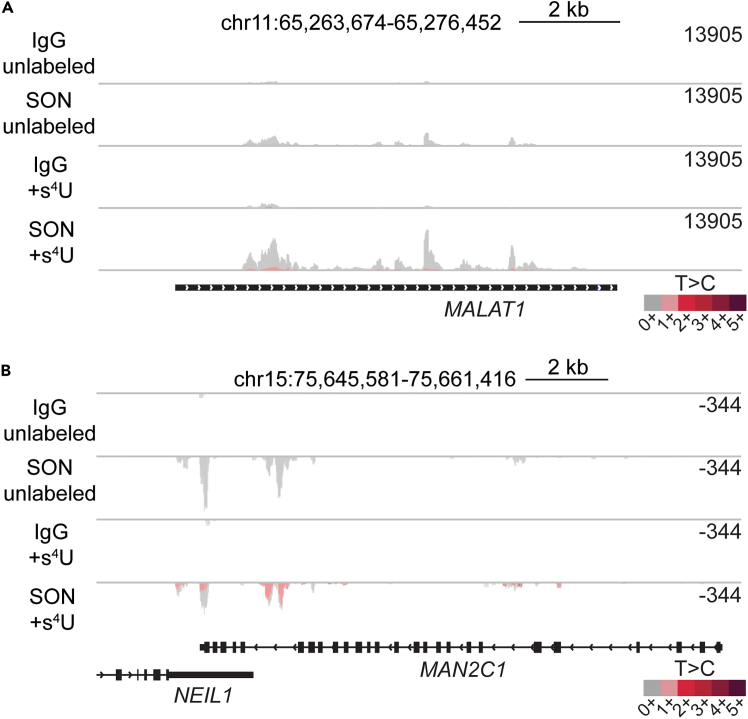
Representative SLAM-RT&Tag signal distribution IGV tracks showing SLAM-RT&Tag signal over the gene bodies of (A) *MALAT1* and (B) *MAN2C1*.

To assess SLAM-RT&Tag signal enrichment, examine read alignments over known nuclear speckle-associated transcripts. MALAT1 (Figure 4A), a long noncoding RNA stably localized to nuclear speckles, should show strong SC35 signal enrichment across its gene body. MAN2C1 (Figure 4B), a transcript transiently localized to nuclear speckles, also shows SC35 signal enrichment, particularly near the 3′ end due to poly(A) tail priming and within introns from internal poly(A) stretches. While MALAT1 enrichment is expected across cell types, MAN2C1 signal may vary by cell type.

## Limitations

SLAM-RT&Tag is an *in situ* method that detects signal from metabolically labeled transcripts near an antibody-targeted nuclear compartment epitope. Consequently, its application is restricted to live cells capable of efficient s^4^U uptake. Certain primary cells or poorly permeable cell lines may exhibit suboptimal s^4^U labeling efficiency, making them unsuitable for SLAM-RT&Tag. Therefore, s^4^U labeling conditions should be empirically optimized for each cell line used.

The method is highly dependent on the quality of two key reagents: the pAG-Tn5 enzyme and the primary antibody. Suboptimal pAG-Tn5 activity can impair tagmentation efficiency, leading to poor library quality and excessive adapter artifacts. Pilot experiments are recommended to batch test pAG-Tn5 activity prior to large-scale experiments. Similarly, poorly characterized or nonspecific antibodies can result in high background and nonspecific signal.

Transcript assignment to nuclear compartments is constrained by the labeling radius of the tethered reagents. While Tn5 transposase is known to tagment DNA within approximately a nucleosome’s distance,^14^ the spatial resolution of SLAM-RT&Tag is less defined. In its current form, SLAM-RT&Tag detects transcripts proximal to the targeted nuclear compartment but cannot distinguish between transcripts that are merely proximal versus those residing directly within the compartment. Future iterations of the method with a reduced labeling radius may resolve this limitation.

Lastly, SLAM-RT&Tag requires high sequencing coverage (>20 million reads) to perform differential kinetic analysis. Lowly expressed transcripts (<50 reads) will fail to meet the read depth required for accurate kinetic modeling by bakR, limiting the ability to assess the dynamics of all compartment-enriched transcripts.

## Troubleshooting

### Problem 1

Beads become excessively sticky before the reverse transcription and tagmentation step, resulting in sample loss (isolation of nuclei from human K562 cells, step 19).

### Potential solution

• Bead stickiness can indicate excessive sample input. Perform a nuclei titration experiment to determine the optimal number of nuclei to use for your specific cell type. In general, do not to exceed 400,000 nuclei per reaction.
• Confirm that Wash buffer was prepared correctly. Low salt concentrations (below 150 mM) can cause beads to clump.

### Problem 2

A primer artifact (∼150 bp peak) more abundant than the SLAM-RT&Tag library (200–700 bp) is observed on the Tapestation (tapestation analysis, step 75).

### Potential solution

• Low nuclei input may result in insufficient RNA template for generating SLAM-RT&Tag libraries. Increase the number of input nuclei, ideally above 100,000 nuclei.
• Low pAG-Tn5 activity can lead to poor tagmentation of RNA-cDNA hybrids. Use freshly prepared or properly stored pAG-Tn5 and test its activity on a control sample before use. Load the pAG-Tn5 with adapters as soon as possible- prolonged storage can impair adapter loading efficiency.
• RNase contamination can degrade RNA and reduce template availability. Ensure all reagents and work surfaces are RNase-free. If purifying pAG-Tn5 from *E.* *coli*, test for co-purification of bacterial RNases.

### Problem 3

Low HISAT2 alignment rate (<40% of uniquely mapped reads) in unlabeled samples or low SLAM-DUNK alignment rate (<40% of filtered reads) in s^4^U labeled samples (deep sequencing and data processing, step 80).

### Potential solution

• Excessive sequencing of adapter artifacts can reduce the proportion of informative reads. Refer to problem 2 for troubleshooting low input, low pAG-Tn5 activity, or RNase contamination.
• Perform an additional round of size selection to remove small fragments (∼150 bp) and re-sequence.

### Problem 4

No enrichment observed over IgG background (deep sequencing and data processing, step 80).

### Potential solution

• Poor primary antibody performance can result in low signal enrichment. Perform an antibody titration experiment to identify the optimal working concentration. Test an alternative antibody, ideally raised in rabbit, as protein A/G has the highest affinity to rabbit IgG.
• High salt concentration in the 300Wash buffer (300 mM) may disrupt weak protein-RNA interactions. Instead, perform pAG-Tn5 binding and subsequent washes in regular Wash buffer (150 mM). If lowering salt concentration, monitor the IgG control for a potential increase in background signal.

### Problem 5

Unable to estimate degradation rate constants (k_deg_) for most compartment-enriched transcripts (deep sequencing and data processing, step 84).

### Potential solution

• Shallow sequencing depth can limit the ability to model transcript kinetics, particularly for lowly expressed transcripts. Increase sequencing depth per sample (>20 million reads) to improve transcript coverage. If average read quality is below ∼38, adjust bam2bakR config file to lower read quality cut-off below 40. Alternatively, loosen the read count threshold in bakR (e.g., from 50 to 20 reads) to include more transcripts in the analysis.
• Insufficient s^4^U labeling can impede accurate kinetic modeling. Extend the s^4^U labeling duration or increase the s^4^U labeling concentration.

## Resource availability

### Lead contact

Further information and requests for resources and reagents should be directed to and will be fulfilled by the lead contact, Dr. Steven Henikoff (steveh@fredhutch.org).

### Technical contact

For inquiries regarding troubleshooting or protocol details, contact Nadiya Khyzha (nkhyzha@fredhutch.org).

### Materials availability

This study did not generate any new unique reagents.

### Data and code availability

All primary sequencing data have been deposited as single-end fastq files in the Gene Expression Omnibus under accession code GSE272219. Code for analyzing RT&Tag and SLAM-RT&Tag datasets is available at https://github.com/nadiyakhyzha/SLAMRTTag and Zenodo (https://doi.org/10.5281/zenodo.15794169).

## Acknowledgments

We thank Doris Xu for cell culture, Christine Codomo for library pooling, and Jorja Henikoff and Matthew Fitzgibbon for sequencing data preparation. This work was supported by the Howard Hughes Medical Institute (S.H.) and an NSERC Postdoctoral Fellowship (N.K.).

## Author contributions

N.K. performed all the experiments and analyzed the data. N.K., K.A., and S.H. wrote this protocol.

## Declaration of interests

N.K., K.A., and S.H. have filed a patent application on related work.
